# Improving the accuracy of genomic prediction in Chinese Holstein cattle by using one-step blending

**DOI:** 10.1186/s12711-014-0066-4

**Published:** 2014-10-14

**Authors:** Xiujin Li, Sheng Wang, Ju Huang, Leyi Li, Qin Zhang, Xiangdong Ding

**Affiliations:** Laboratory of Animal Genetics, Breeding and Reproduction, Ministry of Agriculture of China, National Engineering Laboratory for Animal Breeding, College of Animal Science and Technology, China Agricultural University, Beijing, 100193 China

## Abstract

**Background:**

The one-step blending approach has been suggested for genomic prediction in dairy cattle. The core of this approach is to incorporate pedigree and phenotypic information of non-genotyped animals. The objective of this study was to investigate the improvement of the accuracy of genomic prediction using the one-step blending method in Chinese Holstein cattle.

**Findings:**

Three methods, GBLUP (genomic best linear unbiased prediction), original one-step blending with a genomic relationship matrix, and adjusted one-step blending with an adjusted genomic relationship matrix, were compared with respect to the accuracy of genomic prediction for five milk production traits in Chinese Holstein. For the two one-step blending methods, de-regressed proofs of 17 509 non-genotyped cows, including 424 dams and 17 085 half-sisters of the validation cows, were incorporated in the prediction model. The results showed that, averaged over the five milk production traits, the one-step blending increased the accuracy of genomic prediction by about 0.12 compared to GBLUP. No further improvement in accuracies was obtained from the adjusted one-step blending over the original one-step blending in our situation. Improvements in accuracies obtained with both one-step blending methods were almost completely contributed by the non-genotyped dams.

**Conclusions:**

Compared with GBLUP, the one-step blending approach can significantly improve the accuracy of genomic prediction for milk production traits in Chinese Holstein cattle. Thus, the one-step blending is a promising approach for practical genomic selection in Chinese Holstein cattle, where the reference population mainly consists of cows.

## Background

A reference population with sufficient size is essential in genomic selection (GS) [1-3]. For dairy cattle, in almost all countries with developed dairy industry, thousands of progeny-tested bulls with highly reliable estimated breeding value (EBV) are used to form the national reference population. However, constituting such a reference population is not feasible in some countries, e.g. China, where the number of bulls with highly reliable EBV is limited. As an alternative, cows can be used to form the reference population. Ding et al. [4] investigated the accuracy of genomic prediction using a reference population consisting of cows, and showed that genomic selection using cows is feasible. However, a larger population of reference cows was required to obtain comparable accuracies of genomic prediction than when progeny-tested bulls are used as reference population, because cow EBV are generally less reliable than bull EBV [4]. Further efforts are needed to improve the accuracy of genomic prediction in such a situation.

The term “one-step blending” was used to distinguish it from the original single-step approach using DPR (de-regressed proofs) instead of raw phenotypes [5]. In the present study, we investigated the possible improvements in the accuracy of genomic prediction by applying the one-step blending approach to Chinese Holsteins, for which the reference population consists primarily of cows. In addition, the influence of the relationship between the non-genotyped animals and genotyped selection candidates on the prediction accuracy of one-step blending was also investigated.

## Methods

### Data

The data consisted of 4917 Chinese Holstein cows born from 1998 to 2009 and 240 progeny-tested bulls born from 1984 to 2005, all of which had official EBV on five milk production traits (milk yield, fat yield, fat percentage, protein yield, and protein percentage). These official EBV were obtained based on a multiple-trait random regression test-day model [6]. DRP of all animals were derived from their EBV according to VanRaden and Wiggans [7] and used as response variables for genomic prediction. Reliabilities of the DRP were calculated according to Liu et al. [8]. All animals had reliabilities of DRP greater than 0.40 (for cows) or 0.80 (for bulls). Out of the 4917 cows, 4106 born before 2008, together with the 240 bulls, were taken as the reference population, and the remaining 811 cows born in or after 2008 were used as the validation population.

All individuals in the reference and validation populations were genotyped with the Illumina BovineSNP50 BeadChip (Illumina, San Diego, CA). Missing genotypes of single nucleotide polymorphisms (SNPs) with known chromosomal positions were imputed by BEAGLE [9], and those with unknown chromosomal positions were discarded. After imputation, SNPs with minor allele frequency (MAF) less than 0.01 were removed, leaving 46 422 SNPs for genomic prediction.

To implement the one-step blending approach, all non-genotyped dams and half-sisters of the validation cows that had DRP with reliabilities greater than 0.40, were considered. Of the 811 validation cows, 425 had non-genotyped dams (424 in total) and all had non-genotyped half-sisters (17 085 in total, ranging from 154 to 2672).

Blood samples were collected from Chinese Holstein cattle when the regular quarantine inspection of the farms was conducted. The procedure for collecting the blood samples was carried out in strict accordance with the protocol approved by the Animal Welfare Committee of China Agricultural University (Permit Number: DK996).

### Statistical models

Three methods, GBLUP, the original one-step blending and the adjusted one-step blending, were implemented for genomic prediction of animals in the validation population.

#### GBLUP

The following genomic BLUP model [10] was used to predict genomic breeding values:

$$ \mathbf{y}=1\upmu +\mathbf{Zg}+\mathbf{e}, $$

where **y** is the vector of DRP of the reference animals, **g** is the vector of additive genetic effects, which assumed to follows a normal distribution $\mathrm{N}\left(0,\mathbf{G}{\sigma}_g^2\right),$ with **G** being the genomic relationship matrix constructed using the first method of VanRaden [10], and **e** is the vector of random errors, assumed to follow a normal distribution $\mathrm{N}\left(0,\ \mathbf{D}{\sigma}_e^2\right)$, with **D** being a diagonal matrix with *d*_*ii*_ = 1/*w*_*i*_, where ${w}_i={r}_{DR{P}_i}^2/\left(1-{r}_{DR{P}_i}^2\right)$ (${r}_{DR{P}_i}^2$ is the reliability of DRP of individual *i*) [10,11]. The estimates in **g** based on this model are termed direct genomic breeding values (DGV).

#### Original one-step blending

Following Legarra et al. [12], Aguilar et al. [13], and Christensen and Lund [14], the one-step blending method has the same model as GBLUP, except that the vector **y** also contains the DRP of non-genotyped animals and vector **g** is assumed to follow a normal distribution $\mathrm{N}\left(0,\mathbf{H}{\sigma}_g^2\right)$, where **H** is defined as:

$$ \mathbf{H}=\left[\begin{array}{cc}\hfill {\mathbf{A}}_{11}+{\mathbf{A}}_{12}{{\mathbf{A}}_{22}}^{-1}\left(\mathbf{G}-{\mathbf{A}}_{22}\right){{\mathbf{A}}_{22}}^{-1}{\mathbf{A}}_{12}^{\prime}\hfill & \hfill {\mathbf{A}}_{12}{{\mathbf{A}}_{22}}^{-1}\mathbf{G}\hfill \\ {}\hfill \mathbf{G}{{\mathbf{A}}_{22}}^{-1}{\mathbf{A}}_{12}^{\prime}\hfill & \hfill \mathbf{G}\hfill \end{array}\right], $$

with **A**_11_, **A**_12_, and **A**_22_ sub-matrices of **A** (the pedigree-based relationship matrix), and subscripts 1 and 2 refer to non-genotyped and genotyped animals, respectively. The estimates in **g** based on this model are termed the genomic enhanced breeding values (GEBV).

#### Adjusted one-step blending

To avoid the potential incompatibility in scale between the coefficients of **G** and **A**_22_ involved in the **H** matrix, which could lead to incorrect weighting of the pedigree and genomic information, as pointed out by Forni et al. [15], the **G** matrix was adjusted following Gao et al. [16], i.e.,

$$ {\mathbf{G}}_{\mathrm{a}}=\mathbf{G}\beta +\alpha, $$

where *β* and *α* are obtained from the following equations:

$$ \begin{array}{l}\mathrm{A}\mathrm{v}\mathrm{g}\left(\mathrm{diag}\left(\mathbf{G}\right)\right)\beta +\alpha =\mathrm{A}\mathrm{v}\mathrm{g}\left(\mathrm{diag}\left({\mathbf{A}}_{22}\right)\right),\hfill \\ {}\mathrm{A}\mathrm{v}\mathrm{g}\left(\mathrm{offdiag}\left(\mathbf{G}\right)\right)\beta +\alpha =\mathrm{A}\mathrm{v}\mathrm{g}\left(\mathrm{offdiag}\left({\mathbf{A}}_{22}\right)\right),\hfill \end{array} $$

Where Avg(diag(*)) means the average value of diagonal elements of matrix *; Avg(offdiag(*)) means the average value of non-diagonal elements of matrix *.

The variance components $\left({\upsigma}_g^2\;\mathrm{and}\;{\upsigma}_e^2\right)$ involved in the three models were estimated using AI-REML, as implemented in the software DMU [17].

### Evaluation of the accuracy of genomic prediction

The accuracy of genomic predictions was evaluated as ${\mathrm{r}}_{\mathrm{v}}=\frac{{\mathrm{r}}_{\widehat{g},\mathrm{D}\mathrm{R}\mathrm{P}}}{{\mathrm{r}}_{\mathrm{DRP}}}$ [5], where ${\mathrm{r}}_{\widehat{g},\mathrm{D}\mathrm{R}\mathrm{P}}$ is the correlation between the estimated **g** (DGV or GEBV) and the DRP in the validation population and r_DRP_ is the average of the square root of the reliability of the DRP of the validation cows.

In addition, the theoretical accuracy of the DGV or GEBV was calculated for each individual in the same way as in conventional BLUP, following Henderson [18] from the diagonal of the inverse of the mixed model equation (MME), and the average theoretical accuracy over validation animals was also used to evaluate the accuracy of genomic predictions.

## Results and discussion

As shown in Table 1, for the 811 validation cows, r_v_ and average theoretical accuracies from the original one-step blending increased by 0.12 and 0.02, respectively, compared with the accuracies from GBLUP averaged over the five traits. Accuracies from the adjusted one-step blending approach were almost the same as those from the original one-step blending. Theoretical accuracies were much higher than r_v_, which was also observed in other studies [3,19-21]. The theoretical accuracy may also be overestimated owing to sampling errors in elements of the genomic relationship matrix as pointed out by Goddard et al. [22]. In comparison with GBLUP, the one-step blending approach can significantly improve the accuracy of genomic prediction by incorporating the phenotypes (DRP) of non-genotyped relatives of the selection candidates. However, the adjusted one-step blending did not result in further improvements in accuracy compared with the original one-step blending, probably because the original **G** matrix was little adjusted in our situation, since the estimates of *β* and *α* were 0.992 (close to 1) and 0.017 (close to 0), respectively, while they were 0.859 and 0.298 in the study of Christensen et al. [23]. Similar results were also observed by Gao et al. [16] in the Nordic Holstein population, where the adjusted one-step blending resulted in little improvement in the prediction accuracy and estimates of *β* and *α* were 0.976 and 0.085, respectively.

**Table 1:** **Accuracies of genomic prediction for the validation cows**

**Trait**	**GBLUP**		**Original one-step**		**Adjusted one-step**	
	***r***_***v***_ *	**Theoretical accuracy**	***r***_***v***_ *	**Theoretical accuracy**	***r***_***v***_ *	**Theoretical accuracy**
	**All validation cows**					
Milk yield	0.36	0.73	0.46	0.75	0.46	0.75
Fat yield	0.44	0.73	0.60	0.75	0.60	0.75
Fat %	0.52	0.67	0.60	0.69	0.60	0.69
Protein yield	0.37	0.72	0.51	0.74	0.51	0.75
Protein %	0.51	0.67	0.62	0.69	0.62	0.69
Average	0.44	0.70	0.56	0.72	0.56	0.73
	**386 validation cows with genotyped dams**					
Milk yield	0.34	0.73	0.36	0.73	0.37	0.74
Fat yield	0.42	0.73	0.44	0.74	0.44	0.74
Fat %	0.50	0.66	0.52	0.67	0.52	0.67
Protein yield	0.36	0.72	0.37	0.73	0.37	0.73
Protein %	0.48	0.67	0.50	0.67	0.50	0.67
Average	0.42	0.70	0.44	0.71	0.44	0.71
	**425 validation cows with both non-genotyped dams and half-sisters**					
Milk yield	0.36	0.73	0.55	0.76	0.55	0.76
Fat yield	0.46	0.73	0.73	0.76	0.72	0.76
Fat %	0.54	0.67	0.69	0.70	0.69	0.70
Protein yield	0.39	0.75	0.62	0.76	0.62	0.76
Protein %	0.54	0.67	0.73	0.70	0.73	0.70
Average	0.46	0.71	0.66	0.74	0.66	0.74

*r_*v*_ is equal to the correlation between the estimated breeding and the DRP divided by the average of the square root of the reliability of the DRP in the validation cows.

Among the 811 validation cows, 425 had both non-genotyped dams and half-sisters, while 386 with genotyped dams had only non-genotyped half-sisters. For validation cows with genotyped dams, *r*_*v*_ and the theoretical accuracies obtained from both one-step blending approaches were nearly the same as those from GBLUP (Table 1), while for validation cows with both non-genotyped dams and half-sisters, *r*_*v*_ were improved by 15 to 26 percentage points and 1 to 3 percentage points for the theoretical accuracy, when using the one-step blending approach (Table 1). Again, in all these cases, the adjusted one-step blending did not perform better than the original one-step blending. These results suggest that, compared with GBLUP, improvements in accuracies from the one-step blending approach were almost completely contributed by the non-genotyped dams. To further prove this, we discarded all non-genotyped half-sisters and only included the non-genotyped dams of 425 validation cows in the one-step blending approach. As expected, *r*_*v*_ and the theoretical accuracies of the 425 validation cows from the original one-step blending approach (Table 2) were almost the same as those in the scenario when both non-genotyped dams and half-sisters were included in the one-step blending approach (Table 1). The reason for this is that all non-genotyped half-sisters were daughters of 19 genotyped sires in the reference population and the information from these daughters was part of the DRP of the sires. Therefore, these half-sisters contributed little extra information for genomic prediction.

**Table 2:** **Accuracies of genomic prediction for 425 validation cows when their non-genotyped dams and not their non-genotyped half-sisters were used in the one-step blending approach**

**Trait**	**GBLUP**		**Original one-step**	
	***r***_***v***_ *	**Theoretical accuracy**	***r***_***v***_ *	**Theoretical accuracy**
Milk yield	0.36	0.73	0.54	0.76
Fat yield	0.46	0.73	0.73	0.76
Fat percentage	0.54	0.67	0.70	0.69
Protein yield	0.39	0.75	0.61	0.76
Protein percentage	0.54	0.67	0.74	0.69
Average	0.46	0.71	0.66	0.73

*r_*v*_ is equal to the correlation between the estimated breeding and the DRP divided by the average of the square root of the reliability of the DRP in the validation cows.

## Conclusions

Averaged over the five milk production traits, both one-step blending methods increased r_v_ and the average theoretical accuracy by about 0.12 and 0.02, respectively, compared to GBLUP. However, the adjusted one-step blending did not perform better than the original one-step blending in our situation. In our situation, improvements in accuracies from both one-step blending approaches were almost completely contributed by the non-genotyped dams of the validation animals.
